# Association between smoking status and choroidal thickness in the Korean population: Korea National Health and Nutrition Examination Survey 2017–2021

**DOI:** 10.1371/journal.pone.0358964

**Published:** 2026-09-21

**Authors:** Jae Shin Song, Min Seok Kim

**Affiliations:** Department of Ophthalmology, Seoul National University College of Medicine, Seoul National University Bundang Hospital, Seongnam, Korea; Hangil Eye Hospital / Catholic Kwandong University College of Medicine, KOREA, REPUBLIC OF

## Abstract

Smoking has been suggested to influence choroidal circulation and structure, but population-based evidence regarding its association with choroidal thickness remains limited. This cross-sectional, population-based study investigated the association between smoking status and subfoveal choroidal thickness (SFCT) in a nationally representative Korean population, using data from the Korea National Health and Nutrition Examination Survey (KNHANES) 2017–2021. A total of 22,130 participants from KNHANES who had gradable optical coherence tomography images and without ocular pathologies affecting choroidal thickness were included. Smoking status was categorized as never, former, and current smoking based on standardized KNHANES questionnaires. Weighted mean SFCT across smoking groups was compared, and association between smoking status and SFCT were estimated using survey-weighted multivariate linear regression. Mean SFCT showed a stepwise increase from never to former to current smokers in both eyes (right eye: 297.3 ± 1.3 µm, 307.0 ± 2.1 µm, and 330.6 ± 2.3 µm, respectively; left eye: 297.2 ± 1.3 µm, 305.5 ± 2.2 µm, and 330.1 ± 2.2 µm, respectively; all p < 0.001). In multivariate analysis, current smoking was associated with thicker SFCT (right eye: β = 17.55 ± 3.45 µm, left eye: β = 20.47 ± 3.40 µm; both p < 0.001). Older age, female sex, longer axial length, greater central macular thickness, and the presence of cardiovascular disease were independently associated with thinner SFCT in both eyes (all p < 0.05). Current smoking was independently associated with greater SFCT, providing large-scale population-based evidence of the association between smoking status and choroidal structural changes.

## Introduction

Choroidal thickness is increasingly recognized as a critical biomarker in the pathophysiology of retinal disease. Recent advances have highlighted its role not only in the pachychoroid disease spectrum but also in other major retinal conditions, including age-related macular degeneration (AMD), diabetic retinopathy (DR), and high myopia [1–3]. With the development of enhanced depth imaging and swept-source optical coherence tomography, choroidal thickness can now be assessed with high reproducibility in vivo, enabling large-scale epidemiologic investigations [4].

Choroidal thickness is reported to be influenced by demographic and ocular factors such as age, sex, and axial length, while the impact of lifestyle behaviors remains inconsistently reported [5–9]. Among these, cigarette smoking is a major modifiable risk factor for systemic vascular disease and has been consistently associated with a range of retinal diseases such as AMD and central serous chorioretinopathy (CSC) [10,11]. These associations suggest a potential role in modulating disease pathophysiology through alterations in choroidal structure and function. Prior studies on the effects of smoking on choroidal thickness have reported conflicting results, and evidence remains limited due to small sample sizes and insufficient statistical power [12–14]. Notably, there is a paucity of large-scale study has evaluated differences in choroidal thickness across never-smokers, former smokers, and current smokers.

Given the public health burden of smoking and the emerging recognition of choroidal thickness as a clinically relevant biomarker in ocular disease, clarifying their association is necessary. Accordingly, we investigated the association between smoking status and choroidal thickness in a general adult population using data from the Korea National Health and Nutrition Examination Survey (KNHANES) 2017–2021, a nationally representative dataset, thereby providing robust population-level evidence.

## Materials and methods

### Study population

The KNHANES is an ongoing nationwide surveillance system designed to generate representative statistics on the health status, health-related behaviors, and dietary intake of the Korean population [15,16]. KNHANES employs a complex, stratified, multistage, probability-cluster sampling design with a rolling system to ensure representativeness of the non-institutionalized civilian population. In the 2017–2021 survey cycles, optical coherence tomography (OCT) measurements, including subfoveal choroidal thickness (SFCT), were incorporated into the dataset. For the current analysis, we included participants who had available ophthalmic examination results and who completed the smoking-related questionnaire of KNHANES. Participants were excluded if they had ocular conditions known to affect choroidal thickness, including DR, AMD, epiretinal membrane, macular hole, or retinal vein occlusion. Ethical approval for this study was obtained by the Institutional Review Board of Seoul National University Bundang Hospital (IRB No. X-2510-1000-901).

### Definition of variables

Smoking status was assessed using self-reported questionnaires. Participants were categorized into three groups: never smokers, former smokers, and current smokers. Never smokers were defined as individuals who had smoked fewer than five packs (<100 cigarettes) in their lifetime. Former smokers were defined as individuals who had smoked at least five packs (≥100 cigarettes) in the past but had quit smoking at the time of the survey. Current smokers were defined as those who were actively smoking at the time of the survey. Cumulative smoking exposure was quantified using pack-years, calculated as the average number of cigarette packs smoked per day multiplied by the duration of smoking in years.

Axial length (AL) was measured using an optical biometer (IOL Master 500; Carl Zeiss Meditec, Germany), defined as the distance from the anterior corneal surface to the retinal pigment epithelium (RPE) along the visual axis. SFCT was defined as the distance from the outer border of the RPE to the inner surface of the sclera at the foveal center and was measured manually. Central macular thickness (CMT) was defined as the mean retinal thickness within the central 1-mm subfield of the macular thickness map. Both SFCT and CMT measurements were obtained using a spectral-domain OCT device (Cirrus HD-OCT 500, Carl Zeiss Meditec, Germany) [17].

Participants were classified as having hypertension (HTN) if they had a systolic blood pressure (SBP) ≥140 mmHg, a diastolic blood pressure (DBP) ≥90 mmHg, or a self-reported history of physician-diagnosed HTN. Pre-HTN was defined as an SBP of 120–139 mmHg or a DBP of 80–89 mmHg. Diabetes mellitus (DM) was defined as a fasting plasma glucose level ≥126 mg/dL, current use of antidiabetic medication (insulin or oral hypoglycemic agents), or a self-reported physician diagnosis of DM.

### Statistical analysis

All statistical analyses were performed using survey procedures to account for the complex sampling design and sample weights of KNHANES. The survey design was specified using the primary sampling unit (psu), stratification variable (kstrata), and sample weights (wt_itvex) provided by KNHANES. For the pooled analysis of the 2017–2021 KNHANES cycles, annual examination weights were rescaled to account for the combination of five survey cycles. Because ophthalmic examinations were conducted from April 2017, the 2017 examination weight was multiplied by 0.75/4.75, and the 2018–2021 examination weights were multiplied by 1/4.75 to generate the pooled analytic weight. Differences among smoking status were compared using one-way analysis of variance for continuous variables and the χ² test for categorical variables. Univariate and multivariate linear regression analyses were conducted to evaluate the associations between smoking status and SFCT after adjusting for potential confounders that have been reported to be associated with SFCT, including age, sex, body mass index (BMI), blood pressure status, DM, cardiovascular disease, kidney disease, dyslipidemia, AL, and CMT [2,7,9,18–21]. For current smokers, cumulative smoking exposure was additionally quantified using pack-years, and univariate and multivariate linear regression analyses were performed to assess its association with SFCT. Variables demonstrating a p-value < 0.05 in the univariate analyses were subsequently entered into the multivariate models. For categorical variables with more than two levels, the variable was included into the multivariate model as a whole if at least one category was significantly associated with the outcome. Statistical significance was defined as a two-sided p-value < 0.05. All analyses were conducted using R software (version 4.4.2) with the survey package.

## Results

**Fig 1:**
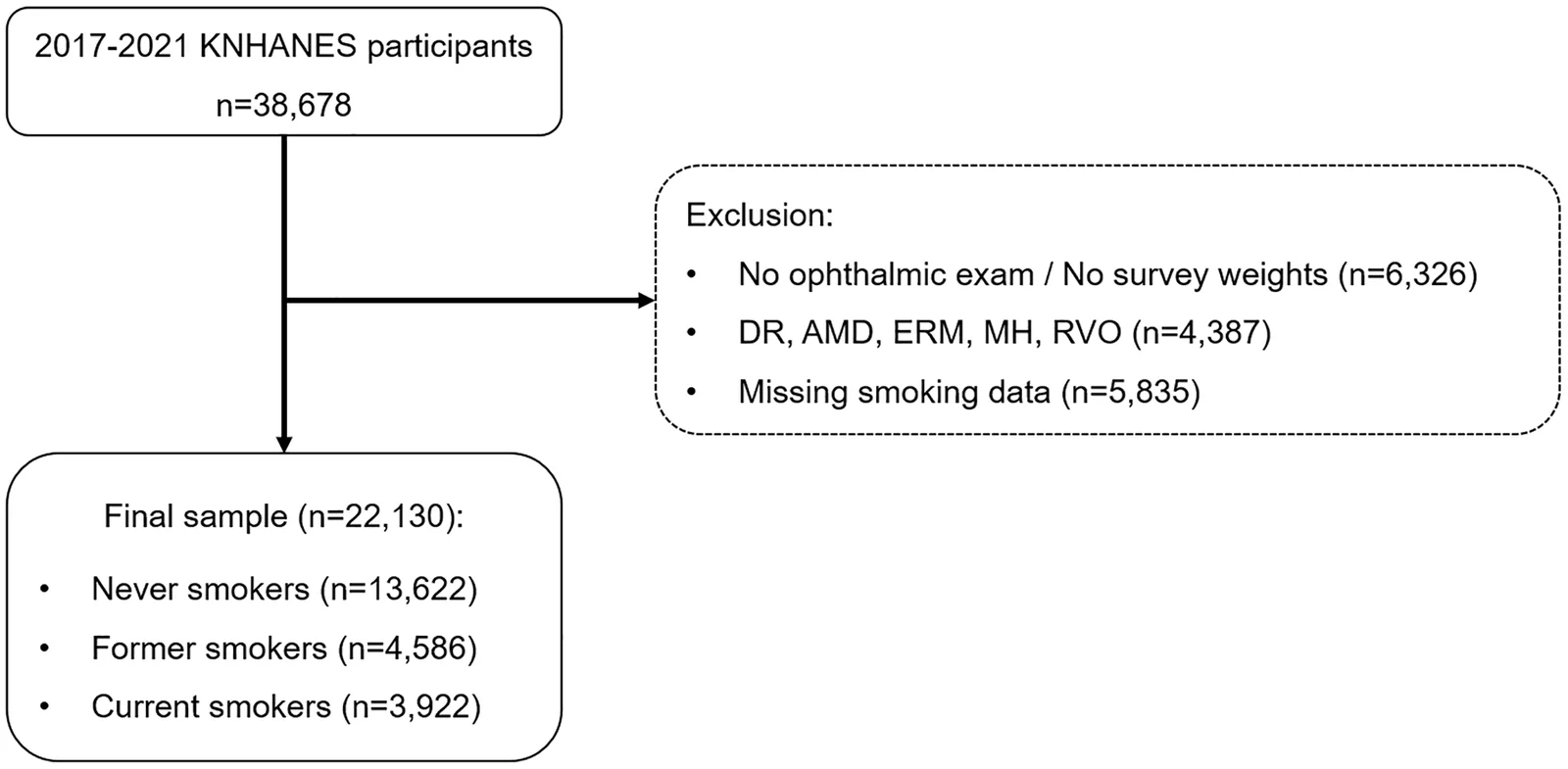
Flow diagram of participant selection from 2017–2021 KNHANES. Among 38,678 participants, individuals were excluded due to lack of ophthalmic examination or survey weights (n = 6,326), presence of ocular diseases including diabetic retinopathy, age-related macular degeneration, epiretinal membrane, macular hole, or retinal vein occlusion (n = 4,387), or missing smoking data (n = 5,835). A total of 22,130 participants were included in the final analytic sample, comprising never smokers (n = 13,622), former smokers (n = 4,586), and current smokers (n = 3,922).

A total of 38,678 participants from the 2017–2021 KNHANES data were initially screened. After exclusion of participants without ophthalmic examinations or with ocular pathologies potentially influencing choroidal thickness, 22,130 individuals were included in the final analysis (Fig 1).

**Fig 2:**
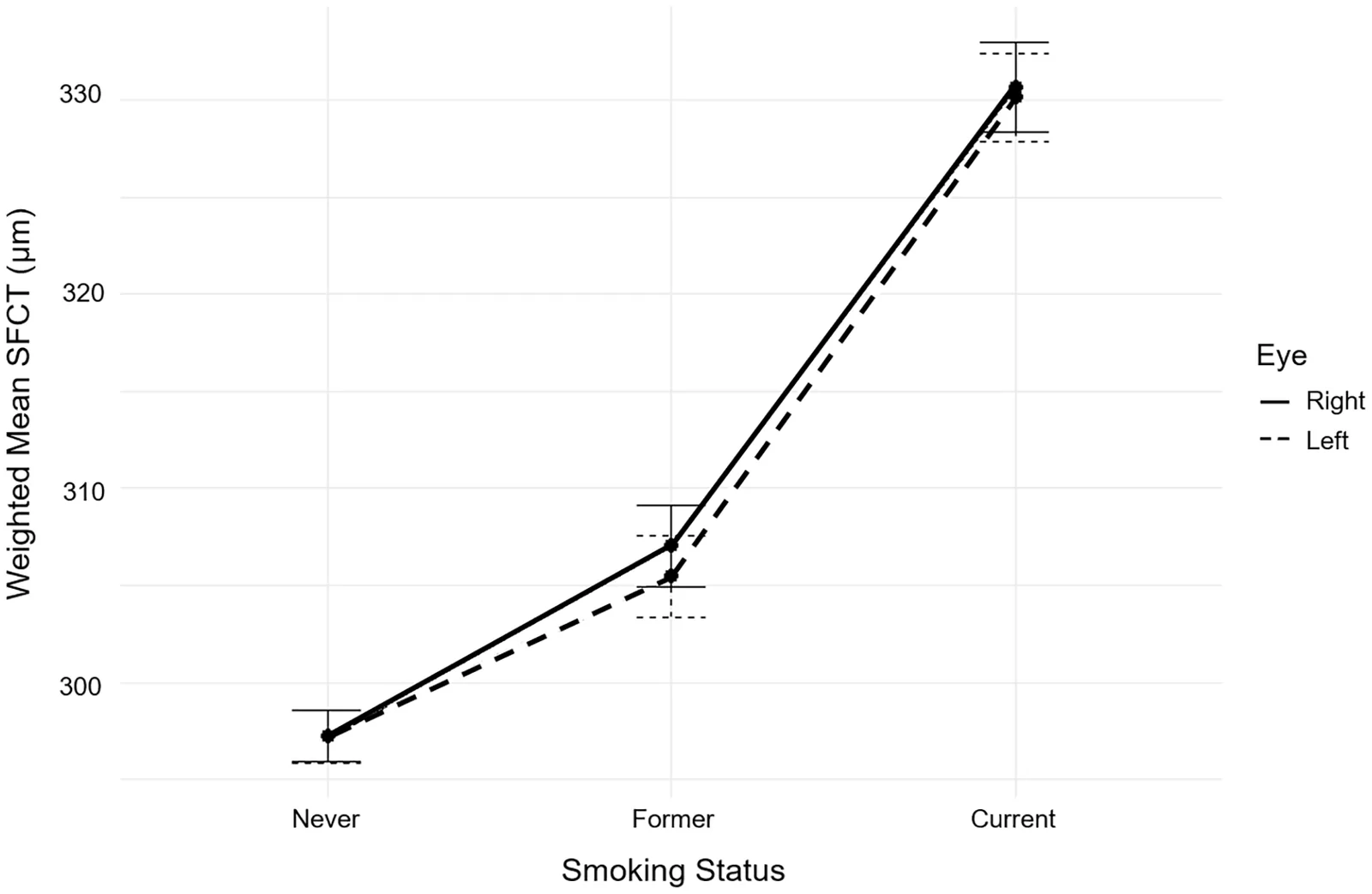
Weighted mean subfoveal choroidal thickness (SFCT) for the right and left eyes is shown across smoking status (never, former, and current smokers). The solid line represents the right eye and the dashed line represents the left eye. Error bars indicate survey-weighted standard errors.

The mean age of participants was 47.0 ± 0.2 years, and 50.1% were women (Table 1). Age and sex distribution differed significantly among the three smoking groups (both p < 0.001). BMI, blood pressure status, DM, cardiovascular disease, and dyslipidemia also showed significant differences across smoking groups (all p < 0.001). Regarding ocular parameters, AL and CMT in both eyes differed significantly among the smoking groups (both p < 0.001). Mean SFCT differed significantly among the groups, showing a progressive increase from never to former to current smokers in both eyes (right eye: 297.3 ± 1.3 µm, 307.0 ± 2.1 µm, and 330.6 ± 2.3 µm, respectively; left eye: 297.2 ± 1.3 µm, 305.5 ± 2.1 µm, and 330.1 ± 2.2 µm, respectively; both p < 0.001). Fig 2 illustrated the weighted mean SFCT according to smoking status, showing a clear stepwise increase from never to former to current smokers in both eyes.

**Table 1:** Demographic and clinical characteristics of study participants by smoking status.

			Total (n = 22,130)	Never smokers (n = 13,622)	Former smokers (n = 4,586)	Current smokers (n = 3,922)	P-value
Age (years)			47.0 ± 0.2	46.8 ± 0.2	51.3 ± 0.3	43.3 ± 0.3	**<0.001**
Sex (female, %)			50.1	76.7	13.3	15.1	**<0.001**
BMI (kg/m^2^)			24.06 ± 0.03	23.67 ± 0.05	24.75 ± 0.06	24.44 ± 0.07	**<0.001**
Blood pressure status (%)		Normal	51.8	53.6	36.4	43.5	**<0.001**
		Pre-HTN	24.9	23.6	28.5	30.9	
		HTN	23.3	22.9	35.1	25.6	
DM (%)			8.3	8.2	14.1	10.2	**<0.001**
CVD (%)			2.0	1.4	3.7	1.8	**<0.001**
Kidney disease (%)			0.8	0.8	0.8	0.8	0.884
Dyslipidemia (%)			33.4	29.8	39.1	39.8	**<0.001**
AL (mm)	Right eye		24.18 ± 0.02	24.07 ± 0.03	24.24 ± 0.04	24.28 ± 0.03	**<0.001**
	Left eye		24.18 ± 0.02	24.07 ± 0.03	24.29 ± 0.04	24.28 ± 0.04	**<0.001**
CMT (µm)	Right eye		248.5 ± 0.3	245.3 ± 0.4	252.9 ± 0.6	252.8 ± 0.6	**<0.001**
	Left eye		248.5 ± 0.3	245.2 ± 0.4	252.6 ± 0.6	253.2 ± 0.6	**<0.001**
SFCT (µm)	Right eye		306.4 ± 1.1	297.3 ± 1.3	307.0 ± 2.1	330.6 ± 2.3	**<0.001**
	Left eye		305.9 ± 1.1	297.2 ± 1.3	305.5 ± 2.1	330.1 ± 2.2	**<0.001**

Values are presented in weighted mean ± standard error or weighted percentage.

AL, axial length; BMI, body mass index; CMT, central macular thickness; CVD, cardiovascular disease; DM, diabetes mellitus; HTN, hypertension; SFCT, subfoveal choroidal thickness.

In univariate analysis, both former and current smokers demonstrated significantly greater SFCT compared with never smokers (right eye: β = 9.78 ± 2.29 µm and 33.37 ± 2.62 µm, respectively; left eye: β = 8.26 ± 2.32 µm and 32.88 ± 2.47 µm, respectively; all p < 0.001) (Table 2). In multivariate analysis, current smoking remained independently associated with thicker SFCT (right eye: β = 17.55 ± 3.45 µm; left eye: β = 20.47 ± 3.40 µm; both p < 0.001). Other independent factors associated with thinner SFCT in both eyes included older age (right eye: β = −2.13 ± 0.12 µm; left eye: β = −2.04 ± 0.16 µm; both p < 0.001), female sex (right eye: β = −22.60 ± 2.98 µm; left eye: β = −18.91 ± 3.32 µm; both p < 0.001), longer AL (right eye: β = −23.32 ± 2.59 µm; left eye: β = −22.04 ± 3.87 µm; both p < 0.001), greater CMT (right eye: β = −0.17 ± 0.05 µm, p = 0.001; left eye: β = −0.15 ± 0.06 µm, p = 0.006), and the presence of cardiovascular disease (right eye: β = −15.50 ± 6.66 µm, p = 0.020; left eye: β = −18.29 ± 7.58 µm, p = 0.016).

**Table 2:** Linear regression analyses of factors associated with subfoveal choroidal thickness according to smoking status.

	Right eye						Left eye					
	Univariate			Multivariate			Univariate			Multivariate		
	β	SE	P value	β	SE	P value	β	SE	P value	β	SE	P value
Smoking (reference = never smokers)												
Former smokers	9.78	2.29	**<0.001**	3.98	2.98	0.182	8.26	2.32	**<0.001**	5.90	3.12	0.059
Current smokers	33.37	2.62	**<0.001**	17.55	3.45	**<0.001**	32.88	2.47	**<0.001**	20.47	3.40	**<0.001**
Age (years)	−1.28	0.08	**<0.001**	−2.13	0.12	**<0.001**	−1.32	0.09	**<0.001**	−2.04	0.16	**<0.001**
Sex (reference = male)	−18.22	1.74	**<0.001**	−22.60	2.98	**<0.001**	−16.19	1.83	**<0.001**	−18.91	3.32	**<0.001**
BMI (kg/m^2^)	0.41	0.27	0.121				0.44	0.27	0.105			
Blood pressure status (reference = normal)												
Pre-HTN	2.67	2.18	0.222	6.52	2.43	**0.007**	1.21	2.19	0.582	4.92	2.37	**0.038**
HTN	−11.11	2.10	**<0.001**	5.06	2.41	**0.036**	−12.85	2.19	**<0.001**	1.64	2.48	0.509
DM	−10.42	2.92	**<0.001**	0.43	3.26	0.896	−10.47	3.01	**0.001**	1.16	3.44	0.736
CVD	−41.58	6.34	**<0.001**	−15.50	6.66	**0.020**	−47.22	6.32	**<0.001**	−18.29	7.58	**0.016**
Kidney disease	−14.81	10.10	0.143				−22.38	9.83	**0.023**	−17.49	12.27	0.154
Dyslipidemia	−1.67	1.93	0.387				−0.06	1.99	0.976			
Axial length (mm)	−16.33	1.52	**<0.001**	−23.32	2.59	**<0.001**	−17.15	2.32	**<0.001**	−22.04	3.87	**<0.001**
CMT (µm)	−0.26	0.05	**<0.001**	−0.17	0.05	**0.001**	−0.18	0.05	**<0.001**	−0.15	0.06	**0.006**

Boldface values were considered significant at p < 0.05.

AL, axial length; BMI, body mass index; CMT, central macular thickness; CVD, cardiovascular disease; DM, diabetes mellitus; HTN, hypertension.

Among current smokers, no significant association was observed between cumulative smoking exposure, as quantified by pack-years, and SFCT in the right eye (β = −0.27 ± 0.19 µm per pack-year, p = 0.159), whereas a weak but statistically significant positive association was noted in the left eye (β = 0.41 ± 0.20 µm per pack-year, p = 0.040) (Table 3).

**Table 3:** Linear regression analyses of associations between cumulative smoking exposure and subfoveal choroidal thickness in current smokers.

	Right eye						Left eye					
	Univariate			Multivariate			Univariate			Multivariate		
	β	SE	P value	β	SE	P value	β	SE	P value	β	SE	P value
Smoking (pack-year)	−0.17	0.15	0.251	0.27	0.19	0.159	−0.12	0.14	0.413	0.41	0.20	**0.040**
Age (years)	−1.56	0.22	**<0.001**	−2.44	0.26	**<0.001**	−1.51	0.22	**<0.001**	−1.89	0.29	**<0.001**
Sex (reference = male)	−13.72	6.16	**0.026**	−36.95	6.73	**<0.001**	−15.84	6.70	**0.018**	−28.89	8.09	**<0.001**
BMI (kg/m^2^)	0.86	0.64	0.180				1.64	0.63	**0.009**	0.41	0.74	0.581
Blood pressure status (reference = normal)												
Pre-HTN	18.18	5.44	**0.001**	18.08	5.50	**0.001**	17.38	5.29	**0.001**	15.52	5.80	**0.008**
HTN	−0.13	5.17	0.980	7.26	5.37	0.177	3.24	5.34	0.544	6.84	6.24	0.274
DM	−5.36	6.29	0.394				−12.68	6.29	**0.044**	−3.25	7.12	0.648
CVD	−39.37	14.85	**0.008**	0.91	15.04	0.952	−49.88	13.45	**<0.001**	−12.29	17.25	0.477
Kidney disease	−15.27	31.33	0.626				−23.4	24.05	0.331			
Dyslipidemia	−4.16	4.80	0.387				0.27	5.01	0.956			
Axial length (mm)	−19.99	3.51	**<0.001**	−25.82	4.48	**<0.001**	−13.96	4.21	**0.001**	−14.92	5.15	**0.004**
CMT (µm)	−0.34	0.10	**<0.001**	−0.14	0.09	0.121	−0.28	0.10	**0.005**	−0.26	0.11	**0.024**

Boldface values were considered significant at p < 0.05.

AL, axial length; BMI, body mass index; CMT, central macular thickness; CVD, cardiovascular disease; DM, diabetes mellitus; HTN, hypertension.

## Discussion

In this nationally representative Korean population, current smoking was independently associated with greater SFCT in both eyes, whereas former smoking showed no significant association after adjustment for confounders. Nevertheless, a stepwise increase in SFCT was observed across smoking status from never to former to current smokers.

Previous studies investigating the relationship between smoking and choroidal thickness have reported conflicting results. Ulaş et al. reported a transient increase in choroidal thickness within 5 minutes after smoking, which returned to baseline by 60 minutes, suggesting an acute vasodilatory response [13]. In contrast, Sizmaz et al. found a significant decrease in choroidal thickness within 1 and 3 hours after smoking, suggesting opposing short-term vasomotor responses [12]. More recently, Alhazmi et al. showed that nicotine exposure alone, whether via gum or electronic cigarettes, did not significantly alter choroidal thickness, indicating that other components of cigarette smoke may contribute to vascular changes [22]. A meta-analysis including 13 observational studies also found no consistent long-term association, although regional differences were noted [14]. Compared with these studies, which were limited to small or short-term cohorts, the present investigation leveraged a large, nationally representative population with clear classification of smoking status into never, former, and current smokers, enabling robust assessment of chronic exposure.

Cigarette smoke induces both sympathetic stimulation through nicotine and vasodilatory effects through carbon monoxide (CO), resulting in transient alterations in choroidal perfusion and vascular tone [23,24]. Moreover, smoking is known to decrease nitric oxide (NO) bioavailability, which impairs endothelium-dependent vasodilation and may lead to compensatory vascular responses in the choroid [25]. These CO- and NO-mediated changes may explain the thicker SFCT observed in current smokers. With continued exposure, chronic smoking may further provoke compensatory vascular remodeling in response to sustained hypoxic stress, inflammation, and endothelial dysfunction, as demonstrated in systemic vascular studies [26,27]. Even after cessation, partial persistence of these vascular adaptations could account for the marginally thicker SFCT observed among former smokers, suggesting a residual effect of prior exposure.

In addition, the present findings align with evidence linking smoking to pachychoroid-spectrum diseases, particularly CSC. Smoking has been consistently identified as a major risk factor for CSC, with proposed mechanisms including sympathetic activation, oxidative stress, and choroidal vascular hyperpermeability [11,28]. Given that CSC is characterized by choroidal thickening and vascular congestion, the greater SFCT observed in current smokers in our study parallels this pachychoroid phenotype.

Among current smokers, cumulative smoking exposure quantified by pack-years showed only a weak positive relationship with SFCT in the left eye, without a clear dose-dependent trend. A ceiling effect may contribute, as choroidal thickening cannot increase indefinitely with greater smoking duration or intensity, potentially blunting the observable dose–response relationship with pack-years. This pattern also suggests that choroidal thickening associated with smoking likely reflects changes that occur in the early phase of smoking exposure rather than slowly progressive structural remodeling with cumulative lifetime exposure.

Several limitations should be acknowledged. First, the cross-sectional nature of the KNHANES data precludes causal inference and limits the ability to assess longitudinal changes in choroidal thickness over time. Second, although major systemic and ocular confounders were adjusted for, unmeasured or unknown factors influencing SFCT may still exist. Third, smoking exposure was self-reported, potentially subject to recall bias. Fourth, the study population was limited to Korean participants, which may restrict the generalizability of our findings to other ethnic populations. Finally, because the timing of OCT imaging could not be standardized, diurnal variation in choroidal thickness and differences in the interval between the last cigarette and image acquisition may have introduced additional variability.

In conclusion, current smoking was associated with greater SFCT in a large, nationally representative Korean population. These findings suggest an association between smoking status and choroidal structural alterations, although longitudinal studies are needed to determine whether these changes contribute to the development or progression of retinal diseases. Recognition of smoking as a modifiable factor associated with choroidal thickness highlights its clinical relevance and reinforces the broader public health importance of smoking cessation.
